# Compliance of Healthcare Providers With the Notifiable Diseases Surveillance System in Riyadh, Saudi Arabia

**DOI:** 10.7759/cureus.41530

**Published:** 2023-07-07

**Authors:** Waleed K Alshamari, Khalid Aldawwas, Mefawez K Al Shammari, Yazeed K Alshammari, Sulaiman I Alsuwailem, Eid H Alkhaldi, Khalid S Almutairi, Salma Alotaby

**Affiliations:** 1 Preventive Medicine, Ministry of Health, Riyadh, SAU; 2 Family Medicine, King Fahad Medical City, Riyadh, SAU; 3 Occupational Therapy, Prince Sultan Military Medical City, Riyadh, SAU; 4 Preventive Medicine, Saudi Public Health Authority, Riyadh, SAU; 5 Nursing, King Saud Medical City, Riyadh, SAU

**Keywords:** notifiable diseases surveillance system, healthcare providers, riyadh, compliance, surveillance system, notifiable diseases

## Abstract

Introduction

Though reporting notifiable diseases is obligatory in Saudi Arabia, and the Saudi Ministry of Health establishes guidelines, there are concerns about healthcare providers’ compliance, and studies evaluating the notifiable diseases surveillance system (NDSS) are lacking, underlying the urgent need to assess the compliance of healthcare providers with the NDSS in Saudi Arabia.

Methods

This cross-sectional study involved doctors, nurses, and epidemiologists working in healthcare facilities in Riyadh, Saudi Arabia. The data collection was done using a self-administered questionnaire. SPSS version 27 software (IBM Corp., Armonk, NY) was used for statistical analyses.

Results

We included 420 participants enrolled in our study, and 63.1% were female. Of 51.4% of participants who worked in private healthcare facilities, 75.7% of them were nurses, while the majority of those working in governmental facilities were doctors (69.1%). The age range was 20-62 years, and the dominant age group was 31-40 years (63.8%). Most participants had no training in epidemiology (79.7%) and of those trained, 64% had a certificate training level. Most notifiable diseases worked were detected in governmental health facilities (35.6% vs. 18.8%). Of those who identified notifiable diseases, 84.3% notified them. COVID-19, measles, and hepatitis A, B, and C were the most notified diseases. The lack of knowledge of the notification system was the most common barrier to the notification among 81 nurses, 39 doctors, and one epidemiologist. There was a significant relationship between being a doctor in the governmental institution and notification timeline (p = 0.024).

Conclusion

This study showed that identifying notifiable diseases was poor despite good compliance among those who identified them. This study showed the lack of proper training of participants, explaining poor knowledge. The findings highlight the differences in notification practices between private and governmental facilities and the need for educational interventions to tackle the knowledge barrier reported.

## Introduction

A part of health information systems, the notifiable diseases surveillance system (NDSS) is a nationwide collaboration that enables all levels of public health to share health information to monitor, control, and prevent the occurrence and spread of state-reportable and nationally notifiable infectious and some non-infectious diseases and conditions [1]. A surveillance system for notifiable diseases may help with public health planning, health promotion, quality improvement, and resource allocation [2,3]. The emergence and spread of infectious and non-infectious diseases can be controlled and prevented using this system.

With the recent coronavirus disease 2019 (COVID-19) pandemic, the importance of NDSS has increased due to the necessity of rapid, accurate, and timely reporting to concerned organizations. Apart from COVID-19, other notifiable diseases include other infectious and contagious diseases [4], whose monitoring and controlling are vital given that these diseases lead to numerous health, financial, and social problems with the risk of overloading the responders and claiming lives [5,6]. A country's ability to control infectious disease outbreaks at its source and stop their spread both inside and outside its borders depends on the effectiveness of its NDSS [7]. One of the barriers to effective NDSS is a lack of reporting knowledge [4,8]. Studies found that most healthcare providers who acknowledged treating patients with notifiable diseases felt like informing public health authorities to comply with mandates instructing providers to report notifiable diseases in many countries, including Saudi Arabia [8-11]. Some studies revealed that healthcare providers do not understand their duty to report or think this responsibility belongs to other healthcare team members [8,11].

A study evaluating the performance of the communicable disease surveillance system at the primary healthcare level in Jeddah, Saudi Arabia, conducted by Alshehri et al. [9] showed that most primary healthcare physicians were well equipped to use the system, but the practice was poor, with limited internet access as a barrier. However, this study did not evaluate their knowledge and compliance.

Healthcare providers' compliance ensures appropriate investigation and control measures by relevant healthcare authorities, and there is a need to explore the NDSS compliance of healthcare providers in Riyadh to accurately inform local health policies to improve the system and ensure effective disease control and management. Therefore, this study examined the compliance of healthcare providers with the NDSS as well as their knowledge and practices in Riyadh.

## Materials and methods

Study design and setting

This cross-sectional study was conducted in hospitals and primary healthcare centers in Riyadh, Saudi Arabia, from March 2023 to June 2023.

All healthcare providers working in hospitals and primary healthcare centers, including physicians, nurses, and epidemiologists, were eligible for our study. Riyadh is the capital of Saudi Arabia and a commercial hub with over 7 million residents as of 2020. It has 47 hospitals or clinics and the highest number of primary healthcare centers in Saudi Arabia (438). Medical and nurse students were excluded.

Sample size and sampling technique

The minimum sample size (n) was calculated considering a 95% confidence level, an assumed proportion of participants for a maximum sample size calculation of 50%, and a 5% margin of error. The minimum calculated sample size was 384 participants. To compensate for possible inaccurate responses and erroneous completeness of questionnaires, we recruited 420 participants.

A stratified multistage random sampling technique was used. In the first stage, healthcare facilities were divided into two strata (government or private). Secondly, each strata section was divided into two parts (hospitals and a complex of clinics or primary healthcare centers). Thirdly, healthcare workers were divided into three categories based on profession type (physicians, nurses, and epidemiologists). Finally, 35 participants were randomly selected from each substratum, making 420 participants in total.

Data collection

We employed a self-administered questionnaire previously used in a similar study conducted in South Africa [7]. The questionnaire has questions on socio-demographics, participants’ compliance, knowledge, and practices toward NDSS, and factors influencing compliance with the NDSS. The questionnaire was adapted to fit our study's objectives and pilot-tested on 40 participants for its clarity and wording. Then the results were used for improvement only. For validation, the questionnaire was reviewed by three experts, and Cronbach's coefficient alpha test was used to measure the questions' internal consistency (reliability) and showed high reliability with a coefficient of 0.89. The questionnaires were distributed online via emails and social media, such as Google Forms, study descriptions, and invitation letters.

Data analysis

The IBM SPSS Statistics software, version 27 (IBM Corp, Armonk, NY), was used for data entry and statistical analyses. Continuous variables were described as mean ± standard deviation (SD), and categorical variables were expressed in frequency (percentage). Pearson’s chi-square test was used to compare response variables between physicians, nurses, and other healthcare providers, and the p-value was set at <0.05 for statistical significance.

Ethical approval

The approval for this study proposal was obtained from the King Fahad Medical City Institutional Review Board (Ref.: 23-132E), and written permission and consent were requested from health facilities and participants, respectively. The anonymity of the questionnaires ensured confidentiality.

## Results

A total of 420 participants were enrolled in our study. More than half of the participants worked in private hospitals (167, 51.4%), and the majority were nurses (138, 68.3%). However, in governmental hospitals, the majority were doctors (123, 63.4%). The minimum age of the participants was 20 years, and the maximum age was 62 years. The most dominant age group was 31-40 years (253, 63.8%), and almost two-thirds of participants were female (250, 63.1%). Among nurses recruited, most were emergency room (ER) nurses (250, 63.1%), followed by outpatient nurses (62, 31.6%), and resident doctors were the majority among doctors (60, 31.1%), followed by specialists (51, 28.6%). Most participants had no training in epidemiology (315, 79.7%), and of those who were trained in epidemiology (49, 64.2%) had a certificate training level. Table 1 shows further socio-demographic details of all participants enrolled in our study.

**Table 1:** Socio-demographic characteristics of the participants

Variables	Private hospital			Government hospital			Total
	Nurse	Doctor	Epidemiologist	Nurse	Doctor	Epidemiologist	
Types of facility							
Primary health care	32 (68.1%)	11 (23.4%)	4 (8.5%)	14 (26.4%)	37 (69.8%)	2 (3.8%)	100 (25.2%)
Polyclinic	41 (62.1%)	21 (31.8%)	4 (6.1%)	1 (20%)	4 (80%)	0	71 (17.9%)
Secondary hospital	26 (66.7%)	13 (33.3%)	0	7 (24.1%)	21 (72.4%)	1 (3.4%)	68 (17.1%)
Tertiary hospital	39 (78%)	11 (22%)	0	46 (43%)	61 (57%)	0	157 (39.6%)
Total	138 (68.3%)	56 (27.7%)	8 (3.9%)	68 (35.1%)	123 (63.4%)	3 (1.5%)	396
Ages							
20-30	27 (71.1%)	7 (18.4%)	4 (10.5%)	14 (23%)	46 (75.4%)	1 (1.6%)	99 (25%)
31-40	97 (70.8%)	38 (27.7%)	2 (1.5%)	45 (38.8%)	70 (60.3%)	1 (0.9%)	253 (63.8%)
41-50	10 (50%)	10 (50%)	0	7 (50%)	6 (42.9%)	1 (7.1%)	34 (8.58%)
51-62	4 (57.1%)	1 (14.3%)	2 (28.6%)	2 (66.7%)	1 (33.3%)	0	10 (2.5%)
Total	138 (71%)	56 (26%)	8 (3%)	68 (35.1%)	123 (63.4%)	3 (1.5%)	396
Gender							
Male	19 (45.2%)	21 (50%)	2 (4.8%)	12 (11.5%)	90 (86.5%)	2 (1.9%)	146 (36.8%)
Female	119 (74.4%)	35 (21.9%)	6 (3.8%)	56 (62.2%)	33 (36.7%)	1 (1.1%)	250 (63.1%)
Total	138 (68.3%)	56 (27.7%)	8 (3.9%)	68 (35.01%)	123 (63.4%)	3 (1.5%)	396
Category of nurse							
ER nurse	38 (29%)			36 (55.4%)			74 (37.7%)
In-patients	36 (27.5%)			22 (33.8%)			58 (29.6%
Out-patients	57 (43.5%)			5 (7.7%)			62 (31.6%)
Infection control	0			1 (1.5%)			1 (0.51%)
Others	0			1 (1.5%)			1 (0.51%)
Total	131 (75.7%)			65 (24.3%)			196
Category of doctors							
Intern		1 (1.8%)			7 (5.7%)		8 (4.4%)
Resident		9 (16.4%)			51 (41.5%)		60 (31.1%)
General practitioner		22 (40%)			12 (9.8%)		34 (18.5%)
Specialist		15 (27.3%)			36 (29.3%)		51 (28.6%)
Consultant		8 (14.5%)			17 (13.8%)		25 (14.04%)
Total		55 (30.89%)			123 (69.1%)		178
Category of specialty							
Preventive medicine		2 (4.1%)			26 (22.4%)		28 (16.9%)
Emergency medicine		9 (18.4%)			8 (6.9%)		17 (10.3%)
Family medicine		7 (14.3%)			22 (19%)		29 (17.5%)
Internal medicine		8 (16.3%)			15 (12.9%)		23 (13.9%)
Obstetrics and gynecology		10 (20.4%)			16 (13.8%)		26 (15.7%)
Ophthalmology		2 (4.1%)			0		2 (1.2%)
Pediatrics		5 (10.2%)			17 (14.7%)		22 (13.3%)
Physical medicine and rehabilitation		2 (4.1%)			5 (4.3%)		7 (4.02%)
Surgery		4 (8.2%)			4 (3.4%)		8 (4.8%)
Neurology		0			1 (0.9%)		1 (0.60%)
Cardiology		0			2 (1.7%)		2 (1.21%)
Total		49 (29.6%)			116 (70.3%)		165
Training on epidemiology							
Yes	23 (79.3%)	4 (13.8%)	2 (6.9%)	12 (23.1%)	39 (75%)	1 (1.9%)	81 (20.3%)
No	115 (66.5%)	52 (30.1%)	6 (3.5%)	56 (39.4%)	84 (59.2%)	2 (1.4%)	315 (79.7%)
Total	138 (68.3%)	56 (27.7%)	8 (3.96%)	68 (35.1%)	123 (63.4%)	3 (1.5%)	396
Level of training							
Certificate	15 (88.2%)	2 (11.8%)	0	9 (28.1%)	22 (68.8%)	1 (3.1%)	49 (642%)
Diploma	3 (100%)	0	0	1 (50%)	1 (50%)	0	5 (6.32%)
Bachelor's degree	7 (100%)	0	0	2 (50%)	2 (50%)	0	11 (13.9%)
Master's degree	0	1 (33.3%)	2 (66.7%)	1 (33.3%)	2 (66.7%)	0	6 (7.59%)
Doctorate	0	0	0	0	8 (100%)	0	8 (10.1%)
Total	25	3	2	13	35	1	79

Table 2 shows participants who diagnosed and suspected notifiable infectious diseases. Only around a quarter (107, 27.02%) were able to diagnose and suspect a notifiable disease. Most participants who diagnosed notifiable diseases worked in governmental health facilities (69, 35.6%), compared to private facilities (18.8%). More doctors in government facilities than in private facilities (41.1% vs. 27.3%) were able to diagnose notifiable diseases. Most participants who diagnosed notifiable diseases notified them (91, 84.3%). While there was no difference between nurses in private and governmental facilities in terms of notification, more doctors in governmental facilities notified the diseases than their counterparts in private facilities. Compared to doctors, more nurses notified the diseases (97.5% vs. 75.6%).

**Table 2:** Participants who detected notifiable diseases

Diagnosed or suspected any notifiable infectious disease in the last year								
	Yes	No	Unsure	Total				
Private	38 (18.8%)	129 (63.9%)	35 (17.3%)	202				
Government	69 (35.6%)	101 (52.1%)	24 (12.4%)	194				
	107 (27.02%)	230 (58.1%)	59 (14.9%)	396				
Nurse	Private				Government			
	Yes	No	Unsure	Total	Yes	No	Unsure	Total
ER nurse	4 (10.5%)	29 (76.3%)	5 (13.2%)	38	11 (30.6%)	22 (61.1%)	3 (8.3%)	36
In-patients	7 (19.4%)	24 (66.7%)	5 (13.9%)	36	4 (18.2%)	15 (68.2%)	3 (13.6%)	22
Out-patients	10 (17.5%)	34 (59.6%)	13 (22.8%)	57	1 (20%)	4 (80%)	0	5
Infection control	0	0	0	0	1 (100%)	0	0	1
Others	0	0	0	0	0	1 (100%)	0	1
Doctors								
Intern	0	1 (100%)	0	1	4 (57.1%)	3 (42.9%)	0	7
Resident	0	7 (77.8%)	2 (22.2%)	9	15 (29.4%)	28 (54.9%)	8 (15.7%)	51
General practitioner	8 (36.4%)	12 (54.5%)	2 (9.1%)	22	6 (50%)	3 (25%)	4 (25%)	13
Specialist	4 (26.7%)	8 (53.3%)	3 (20%)	15	15 (41.7%)	16 (44.4%)	5 (13.9%)	36
Consultant	3 (37.5%)	3 (37.5%)	2 (25%)	8	11 (64.7%)	5 (29.4%)	1 (5.9%)	17
Total	15 (27.3%)	31 (56.4%)	9 (16.3)	55	51 (41.1%)	55 (44.3%)	17 (13.7%)	124
Did you notify the infectious disease?								
Private	33 (86.8%)	1 (2.6%)	4 (10.5%)	38				
Government	58 (82.9%)	8 (11.4%)	4 (5.7%)	70				
Total	91 (84.3%)	9 (8.3%)	8 (7.4%)	108				
Nurse	Private hospital				Government hospital			
	Yes	No	Unsure	Total	Yes	No	Unsure	Total
ER nurse	4 (100%)	0	0	4	9 (81.8%)	0	2 (18.2%)	11
In-patients	6 (85.7%)	0	1 (14.3%)	7	4 (100%)	0	0	4
Out-patients	10 (100%)	0	0	10	1 (100%)	0	0	1
Infection control	0	0	0	0	1 (100%)	0	0	1
Doctors	Yes	No	Unsure	Total	Yes	No	Unsure	Total
Intern	0	0	0	0	2 (50%)	0	2 (50%)	4
Resident	0	0	0	0	12 (75%)	4 (25%)	0	16
General practitioner	7 (87.5%)	0	1 (12.5%)	8	6 (100%)	0	0	6
Specialist	3 (75%)	0	1 (25%)	4	11 (73.3%)	4 (26.7%)	0	15
Consultant	1 (33.3%)	1 (33.3%)	1 (33.3%)	3	11 (100%)	0	0	11

**Figure 1:**
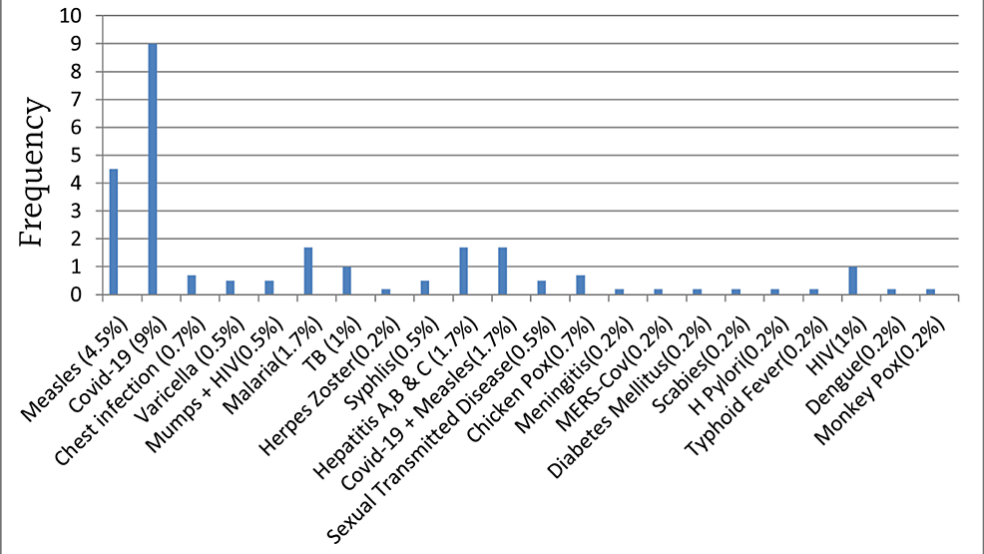
Notifiable infectious diseases diagnosed by the participants

The most notifiable diseases diagnosed were COVID-19, measles, and hepatitis A, B, and C, as shown in Figure 1.

Table 3 shows barriers to notification reported by all participants (nurses, doctors, and epidemiologists) in private and governmental facilities. The lack of knowledge of the notification system was the most reported barrier by nurses (n = 81), doctors (n = 39), and epidemiologists (n = 1). Most of those who notified within four hours were government doctors (n = 20), and there was a significant relationship between being a doctor in the governmental institution and notification timeline (p = 0.024). The participants with the highest NDSS skills (with the highest NDSS scores of 61-100) were private nurses (n = 82), followed by governmental doctors (n = 60). There was no significant correlation between the job types of participants and the barriers or skills (p > 0.05).

**Table 3:** Barriers to notification of notifiable diseases diagnosed, time to notification after diagnosis, and level of skills to perform NDSS tasks NDSS: notifiable diseases surveillance system.

		Lack of knowledge/awareness	Fear of discrimination	Privacy concern	Denial or avoidance	Lack of trust	Total	P-value
Nurse	Private	55	6	10	4	5	80	0.156
	Government	34	1	6	0	0	41	
		81	5	15	3	4	121	
Epidemiology	Private	1	0	1	0	0	2	0.233
	Government	0	0	0	0	1	1	
		1	0	1	0	1	3	
Doctor	Private	17	2	6	0	4	29	0.069
	Government	22	2	13	3	0	40	
		39	4	19	3	4	69	
		Immediately (first 4 hours)	Daily (within 24 hours)	Weekly	Monthly	Total		
Nurse	Private	13	6	2	0	21		0.350
	Government	12	3	0	0	15		
Epidemiology	Private	2	0	0	0	2		0.083, 0.024
	Government	0	1	0	0	1		
Doctor	Private	3	4	1	2	10		
	Government	20	20	2	0	42		
NDSS score out of 100		Low skills (NDSS score) 0-50	Average skills (51-60)	High skills (61-100)				
Nurses	Private	36	20	82				0.147
	Government	13	17	38				
Epidemiology	Private	1	0	7				0.131
	Government	1	1	1				
Doctor	Private	24	10	22				0.302
	Government	59	13	60				
Extent the following interventions would benefit the notifiable diseases surveillance system								
		(1-10) No benefit	(11-30) Average	(31-50) Max benefit		P-value		
Nurses	Private	0	51	87		0.128		
	Government	2	25	41				
Epidemiology	Private	2	0	6		0.190		
	Government	1	1	1				
Doctor	Private	2	24	30		0.451		
	Government	4	41	78				
	Total	11	142	243				

**Figure 2:**
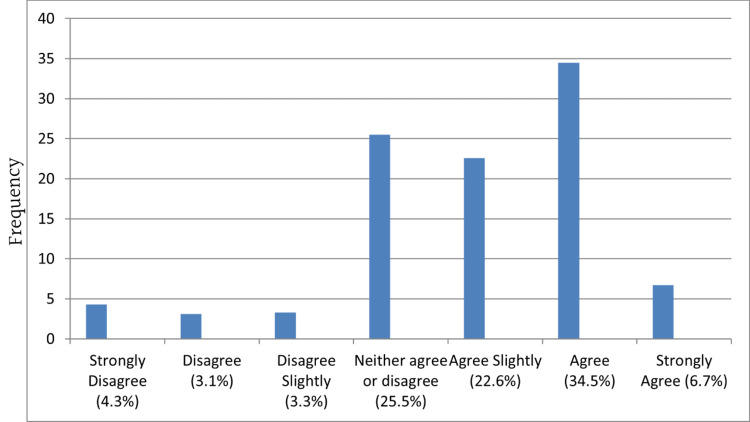
The form used to report notifiable diseases is easy to understand

**Figure 3:**
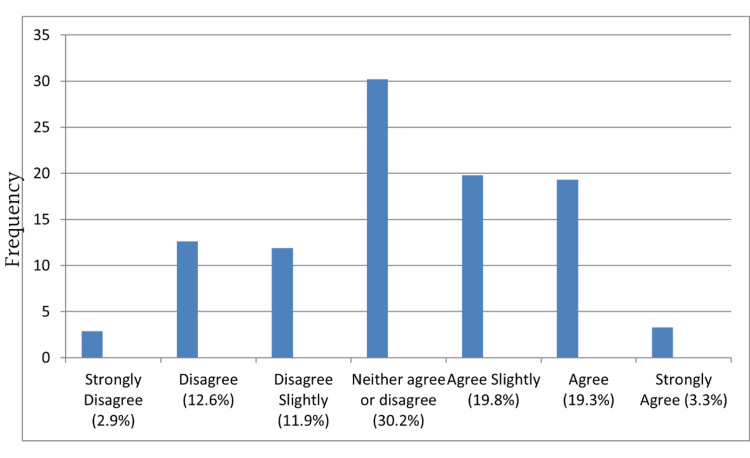
The notification process is not easy to comply with

**Figure 4:**
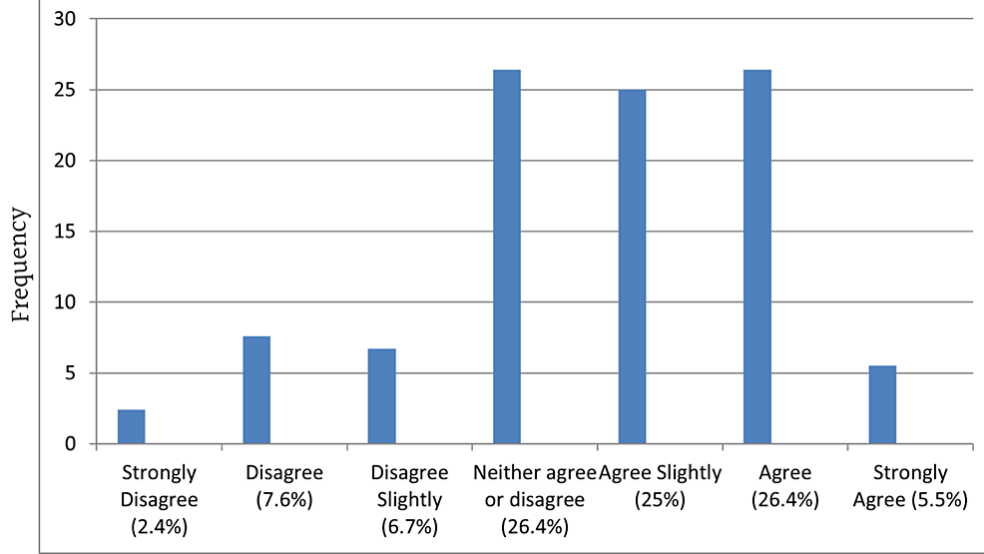
The form used to notify diseases takes a long time to fill in

Most agreed that the notification form was easy to understand (Figure 2). However, more agreed that the notification process is not easy to comply with (Figure 3) and the form used to notify diseases takes a long time to fill in (Figure 4) than those who disagreed.

When asked to rate the availability level of investment to fund the NDSS and organizational capacity for the NDSS, most participants from the private sector rated the NDSS staffing, funding, and organization capacity as very good at national, provincial, district, and facility levels. However, most participants from the governmental facilities rated the NDSS staffing, funding, and organization capacity as satisfactory at national, provincial, district, and facility levels (Table 4).

**Table 4:** Investment of funding and organizational capacity for the NDSS NDSS: notifiable diseases surveillance system.

Availability level of investment of funding for the notifiable diseases surveillance system level										
	Private						Government			
	National	Province	District			Facility	National	Province	District	Facility
Very poor	5	3	4			4	13	15	14	12
Poor	4	8	12			10	13	17	19	15
Satisfactory	58	74	74			67	73	81	74	60
Good	36	41	45			46	61	46	55	45
Very good	99	76	67			75	34	35	32	26
Level of investment of funding for the notifiable diseases surveillance system										
	Private						Government			
	National	Province	District		Facility		National	Province	District	Facility
Very poor	3	2	2		3		10	10	11	11
Poor	9	9	11		9		18	13	15	15
Satisfactory	65	71	74		73		78	87	86	79
Good	37	54	43		47		50	55	51	58
Very good	88	66	72		70		38	29	31	31
Rate the organizational capacity for the notifiable diseases surveillance system										
	Private						Government			
	National	Province	District	Facility			National	Province	District	Facility
Very poor	5	4	3	4			16	12	11	11
Poor	5	10	6	8			21	16	17	14
Satisfactory	68	70	73	73			64	89	83	90
Good	46	52	60	47			52	43	49	45
Very good	78	66	60	70			41	34	34	34

## Discussion

The surveillance and reporting of notifiable diseases play a crucial role in protecting public health. The NDSS enables health authorities to monitor disease patterns, implement targeted control measures, and allocate resources efficiently [12]. In Saudi Arabia, the Ministry of Health has implemented a comprehensive system to monitor and control the spread of such diseases [5,13]. However, the effectiveness of this system depends on how well healthcare providers comply with reporting and managing notifiable diseases. Therefore, this study assessed the level of compliance among healthcare providers with the NDSS knowledge and practices in Saudi Arabia.

We found that the diagnosis/detection of notifiable diseases was poor among our study’s participants, with only a quarter reporting to have diagnosed them, and the diagnosis level was even lower in private facilities. In contrast, the study exploring compliance of 919 healthcare providers with the NDSS in South Africa showed that 58% had identified notifiable diseases, though 51% of those identified were accurate, with the lowest accuracy among pediatricians (OR = 0.01, 95% CI = 0.00-0.12, p = 0.001) [7]. A survey conducted in six Nigerian cities also found a higher diagnosis rate than in our study. It indicated that 66.5% of doctors identified a notifiable disease [4]. In the United States (US), a study conducted by Fill et al. [8] at Vanderbilt University Medical Center indicated that 82% of healthcare providers acknowledged they cared for patients with reportable diseases, and 98.4% believed that they were responsible for reporting to health authorities [8]. Our study showed that most participants who identified the notifiable diseases worked for the government. This may be attributed to a large number of patients, mostly with socio-economic constraints, who consult public health facilities that work with their insurance scheme (universal coverage) [14,15]. This group of people is highly prone to notifiable infectious diseases; most are managed at the primary healthcare centers, which are public in the majority [9].

Detecting and reporting these diseases on time is vital for initiating appropriate public health interventions and preventing further transmission. Some studies have reported poor reporting habits among healthcare providers, contrasting our study showing that most participants (84.3%) who identified notifiable diseases reported them. However, less than 92% of healthcare providers reported them in South Africa [7]. In the US, the reporting was even lower since only 47.2% of those who identified notifiable diseases had ever reported notifiable diseases [8].

Our findings showed higher detection and notification of notifiable diseases among nurses, which might be because nurses are the first to meet patients and screen them, increasing their likelihood of detecting the diseases to notify. In addition, we found higher NDSS skills among nurses, especially in the private sector, which might result in higher detection and notification. These findings align with a study conducted in Egypt, showing that head nurses had better knowledge than physicians [11]. In Saudi Arabia, if the nurse suspected infectious diseases during triage, they should isolate the suspected patients, and then inform a doctor or infection control authority. The sample of the suspected patient is then sent for testing and confirmation. Later, the diagnosis is notified to the NDSS. Therefore, nurses who are at the frontline, screening patients as they come, are more likely to detect the notifiable disease.

We found that COVID-19 and measles were the most notified diseases. This may be due to the period in which this study was conducted, marked by the COVID-19 pandemic and measles outbreaks. This study asked participants to mention the diseases they notified in the past year, which coincide with the COVID-19 pandemic period that started in early 2020 and continued till early 2023 [5], and the measles outbreak in a detention center in Makkah, Saudi Arabia, in late 2021 [16]. This outbreak in a well-recognized and the most tourist city put the whole health system on high alert in addition to efforts that were already in place to eradicate COVID-19, which might lead to high notification of these diseases.

Although our findings may explain the significant progress made in Saudi Arabia's NDSS, challenges persist, which might explain the lack of 100% compliance. Several factors contribute to compliance levels, including awareness, training, reporting mechanisms, and coordination between healthcare facilities and public health authorities. We found that the lack of knowledge of the notification system was the most reported barrier to notification, followed by privacy concerns. Other previous studies also reported a lack of knowledge [4,8,10]. Studies conducted in Syria and Jordan reported a high workload, lack of training, and limited internet access as main barriers [9,17]. Public healthcare facilities usually have limited resources and a high workload compared to private facilities, which might contribute to less compliance, as our findings showed less detection and notification among participants from governmental health facilities [18,19]. This is supported by our findings that most participants from the governmental facilities rated the NDSS staffing, funding, and organization capacity as satisfactory, while most participants in private facilities rated them as very good. Other factors influencing compliance include willingness to notify, knowledge of what to notify, possession of notification forms, and understanding of the purpose and importance of the NDSS [20-22]. Patient privacy and data confidentiality concerns can discourage healthcare providers from reporting notifiable diseases. It is crucial to assure healthcare providers that the information they provide will be securely handled and in compliance with relevant data protection laws. This would remove privacy concerns reported by participants of our study.

Improving awareness among healthcare providers about notifiable diseases and the importance of timely reporting is crucial. Regular continuing education programs, workshops, and training sessions should be conducted to keep healthcare providers updated on the latest guidelines and protocols for disease surveillance and reporting. This is supported by research showing that educational interventions increase healthcare workers' knowledge, awareness, and willingness to report notifiable diseases [2,4]. Our findings highlighted the need for these interventions, showing that most participants had no training in epidemiology, which is essential in disease control. Of those trained, the majority had only certificate training levels. Therefore, continuous and advanced training would help improve knowledge, remove barriers to notification, and lead to an effective system. The success of NDSS depends not only on the attitudes of healthcare providers but also on the knowledge and skills that can be gained through training. An Egyptian study reported that despite 75% of healthcare providers having positive attitudes, poor knowledge of how the system works, notifiable diseases to report, and who to report to were major limitations [11]. Simplifying the reporting process through user-friendly electronic systems can encourage healthcare providers to report notifiable diseases promptly. Integration of reporting platforms with existing electronic medical records can streamline the process and reduce the burden on healthcare providers. Additionally, providing feedback on reported cases, acknowledging receipt, and offering updates can enhance provider engagement. Studies conducted in the USA, Australia, and Latin America, found that feedback significantly influenced compliance with the NDSS [20-22].

Despite reports that the notification system is easy to understand, most participants agreed that the notification form takes a long time to fill out and is not easy to comply with. The ease and efficiency of reporting mechanisms influence compliance. Simplifying the reporting process through user-friendly electronic systems can encourage healthcare providers to report notifiable diseases promptly [23]. Integration of reporting platforms with existing electronic medical records can streamline the process and reduce the burden on healthcare providers [24,25].

This study has some limitations to be considered. This study was conducted in one city, which might impact the generalization of its results in other cities of Saudi Arabia. The cross-sectional design used in this study cannot identify causality and is prone to recall bias. Additionally, we did not study paramedics and other professionals in allied health fields who might also play a role in disease notification. Therefore, we recommend extensive longitudinal studies involving multiple cities and rural areas, as well as all professionals in the healthcare sector, to give more insights regarding healthcare providers' compliance with the NDSS in Saudi Arabia.

## Conclusions

Ensuring compliance among healthcare providers with the NDSS is vital for effectively controlling and preventing diseases in Saudi Arabia. This study showed high compliance despite a low detection of notifiable diseases. By addressing key weakness areas such as knowledge and awareness, reporting mechanisms, data privacy concerns, and training, the Ministry of Health can improve compliance. The healthcare authorities in Saudi Arabia should focus on capacity building in governmental facilities to improve their notification performances by increasing staffing and financial investment in NDSS and organizations. Recognizing and rewarding healthcare providers who demonstrate exceptional compliance with the NDSS can be a positive reinforcement. Publicly acknowledging their efforts, offering incentives like continuing education credits or financial rewards, and incorporating compliance metrics into performance evaluations can motivate them to actively participate in disease surveillance and reporting. Continued efforts to strengthen the system and foster collaboration between healthcare providers and public health authorities are recommended to contribute to the overall well-being of the population and the successful management of notifiable diseases.
